# Discovering potential serological biomarker for chronic Hepatitis B Virus-related hepatocellular carcinoma in Chinese population by MAL-associated serum glycoproteomics analysis

**DOI:** 10.1038/srep38918

**Published:** 2017-01-12

**Authors:** Tianhua Liu, Denghe Liu, Riqiang Liu, Hucong Jiang, Guoquan Yan, Wei Li, Lu Sun, Shu Zhang, Yinkun Liu, Kun Guo

**Affiliations:** 1 Liver Cancer Institute, Zhongshan Hospital, Fudan University, Key Laboratory of Carcinogenesis and Cancer Invasion, Ministry of Education, Cancer Research Center, Institutes of Biomedical Sciences, Fudan University, Shanghai, China; 2 Department of Clinical Laboratory, First Affiliated Hospital of Guangxi Medical University, Nanning, Guangxi, China; 3 People’s Hospital of Gangxi Zhuang Autonomous Region, Nanning, Guangxi, China

## Abstract

The accuracy of current biomarkers for the diagnosis of hepatocellular carcinoma (HCC), especially chronic Hepatitis B Virus (HBV)-related HCC, is limited. Recent progress in glycoproteomics has provided a novel platform for screening novel serological biomarkers of HCC. In this study, lectin affinity chromatography by Maackia amurensis lectin (MAL) and iTRAQ combined with mass spectrometric analysis were performed to enrich and identify the glycoprotein fractions in serum samples from HBV-related HCC patients and from healthy controls. Seventeen differential MAL-associated glycoproteins were identified. Among them, Galectin 3 binding protein (Gal-3BP) was selected for further evaluated by ELISA analysis and showed a high diagnostic potential of HBV-related HCC, with the AUC of 0.898 and a sensitivity, specificity and accuracy of 80.00%, 93.75% and 86.88%, respectively. Moreover, we constructed a predictive model through the combined use of serum Gal-3BP and Alpha Fetoprotein (AFP), which improved the sensitivity (from 87.5% to 95%), specificity (from 93.75% to 95%) and accuracy (from 90.63% to 95%) of diagnosing early HCC. These data suggested serum Gal-3BP level is a promising biomarker to identify HBV-related HCC and the combined use of serum Gal-3BP and AFP improves the diagnostic potential of HBV-HCC compared with AFP alone in current clinical practice.

Liver cancer is one of the most common types of cancer in the world, and as a major primary liver cancer, hepatocellular carcinoma (HCC) accounts for 70–85% of total known liver cancers^1^. HCC is most prevalent in developing countries in Southeast Asia and sub-Saharan Africa, where hepatitis B virus (HBV) infection is highly endemic^2^. Unfortunately, the current diagnosis for HBV-related HCC is far from satisfactory; most patients are diagnosed at late stages, and the 5-year survival rate has remained below 12%^2^.

Glycosylation, which is one of the most important post-translational modifications (PTMs) of protein, is widespread in nature, and as a recognition signal, it plays pivotal roles in cell-cell communication, receptor-ligand interactions, signal transduction, and endocytosis. Altered glycosylation patterns could significantly regulate the structure and function of glycoproteins; furthermore, alterations in glycosylation patterns are associated with a variety of physiological and pathological states^3^,^4^. An increase in the number of alterations in glycosylation patterns relative to the normal rate of variation in glycosylation patterns have been described in different types of diseases, such as neurodegenerative diseases (NDs) and cancers^5^. Some specific structures, including core fucosylated N-glycans and sialic acids, have been observed to increase in various cancers including HCC and have been associated with a poor prognosis^6^,^7^,^8^. Aberrant sialylation and enhanced activity of the sialyltransferases have been proven to be characteristic features of cancer cells^9^. Furthermore, Sialyl Lewis^x^, an important sialic acid-containing carbohydrate epitope, and increased α2,3 SialylT activity are involved in the adhesion and metastasis of cancer cells^10^,^11^. Therefore, exploration and identification of specific alternations in glycosylation patterns, such as sialylation, should be an urgent and promising direction in the cancer biomarker research field, including work on HCC biomarkers.

It is worth noting that most of the currently used cancer biomarkers, such as carbohydrate antigen (CA)15-3, CA19-9, CA125, carcino-embryonic antigen (CEA), and Alpha Fetoprotein (AFP), are glycoproteins. More remarkably, as a heterogenetic glycoprotein of AFP, the lens culinaris agglutinin-reactive fraction of AFP (AFP-L3) has been approved by the US Food and Drug Administration (FDA) as a diagnostic index for HCC^12^ and has been increasingly widely used over the past decade. Serum is the most available sample to be the primary clinical specimen in disease diagnosis and biomarker discovery because various glycoproteins from cell surfaces or tissue are released into serum^13^. However, most of the current studies are focused on glycosylation analysis of global serum glycoproteins; in contrast, there are fewer studies on the serum glycoproteins with specific carbohydrate structure. Lectins, which are widely used in glycan research, could recognize glycoprotein fractions by their strong affinity for specific carbohydrate epitopes of glycoproteins, and lectin affinity chromatography is one of the most widely used tools to purify specific glycoproteins from complex mixtures prior to their identification by mass spectrometry (MS)^14^,^15^. Additionally, the latest advances in MS technology are pushing proteomics toward the analysis of post-translational modifications and have made major strides in glycoprotein identification^16^,^17^.

In this study, we enriched glycoprotein fractions in serum samples from Chinese patients with chronic HBV infection and early HCC and in serum samples from healthy controls; the serum samples were enriched by lectin affinity chromatography with Maackia amurensis lectin (MAL), which could bind with the Siaα2,3 Gal structure. The enriched fractions were labeled with mass-balanced isobaric tags (isobaric tag for relative and absolute quantitation, iTRAQ) and were identified by Nano-high-performance liquid chromatography with tandem mass spectrometric (Nano-HPLC-MS/MS) analysis to provide a novel variety of serological biomarker candidates for diagnosing HBV-related HCC. The differentially expressed MAL-associated serum glycoproteins were further validated by western blotting. Among these candidates, based on enzyme-linked immunosorbent assay (ELISA) analysis, Galectin-3-binding protein (Gal-3BP) was verified as one of the potential serological biomarkers for diagnosing HBV-related HCC; moreover, when combined with serum AFP, the diagnostic accuracy of Gal-3BP was enhanced.

## Results

### Identification and relative quantification of MAL-associated serum glycoproteomics from HBV-related HCC patients and from healthy controls

**Figure 1:**
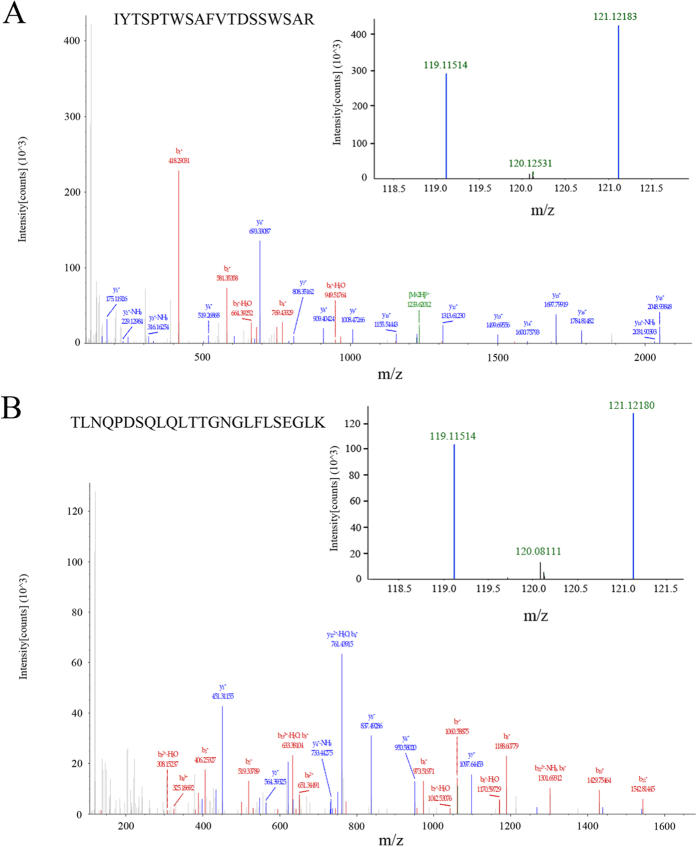
MAL-associated proteins were labeled with an iTRAQ^TM^ Reagent Kit and were examined by Nano-HPLC-MS/MS. (**A**) MS spectra of peptide IYTSPTWSAFVTDSSWSAR digested from Galectin-3-binding protein; the eluted fraction in serum samples from 15 Chinese early HCC patients with chronic HBV infection was labeled by 119 isobaric tag, and the eluted fraction in serum samples from 15 healthy controls was labeled by 121 isobaric tag. (**B**) MS spectra of peptide TLNQPDSQLQLTTGNGLFLSEGLK digested from α-1-antitrypsin.

The same volume of pooled albumin- and IgG-depleted serum samples from 15 Chinese early-stage HCC patients with chronic HBV infection and from 15 healthy controls were enriched by MAL-agarose and labeled with iTRAQ tags. The two groups of peptides were mixed and analyzed by nano-HPLC-MS/MS for protein identification and relative quantification. The identified proteins were further searched using the Uniport database, and 17 MAL-associated serum glycoprotein groups were identified and relatively quantified as differentially expressed glycoproteins by iTRAQ analysis (Table 1). When the ratio of quantified glycoproteins of HBV-related HCC patients to healthy controls was defined as >1.2 or <0.8, eight of them, such as Gal-3BP, α-2-macroglobulin and α-1-antitrypsin, were up-regulated, and the other nine, such as Complement factor B and Vitamin D-binding protein, were down-regulated. Figure 1A,B show representative MS spectra of peptide IYTSPTWSAFVTDSSWSAR digested from Galectin-3-binding protein and TLNQPDSQLQLTTGNGLFLSEGLK digested from α-1-antitrypsin, respectively.

### Validation of the differentially expressed MAL-associated serum glycoproteins

**Figure 2:**
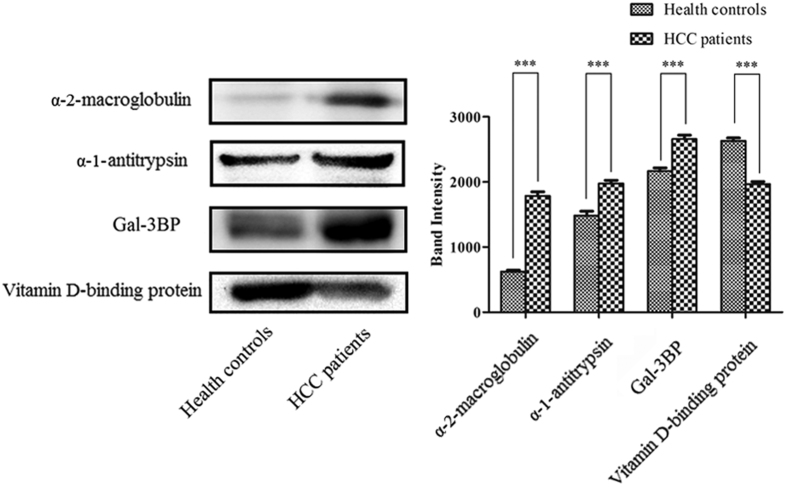
Verification the expression of the secreted differentially expressed MAL-associated serum glycoproteins. Western blotting detection was used to validate the differentially expressed MAL-associated glycoproteins (α-2-macroglobulin, α-1-antitrypsin, Gal-3BP and vitamin D-binding protein) in other pooled serum samples from 15 Chinese HCC patients with chronic HBV infection and from 15 healthy controls. The cropped blots are displayed: all of the four glycoproteins were completely coincident with the iTRAQ quantification trend. Bar graph of α-2-macroglobulin, α-1-antitrypsin, Gal-3BP and vitamin D-binding protein based on quantitative analysis of immunoblotted bands performed by Quantity One v4.62. ***p < 0.001.

To confirm these differentially expressed MAL-associated serum glycoproteins, three up-regulated proteins (Gal-3BP, α-2-macroglobulin and α-1-antitrypsin) and one down-regulated protein (vitamin D-binding protein) were selected for further verification by western blotting in another pool of serum samples from 15 Chinese early HCC patients with chronic HBV infection and 15 healthy controls. Quantitative analysis of immunoblotted bands of these proteins was performed by Quantity One v4.62 (Bio-Rad, Hercules, CA, USA). Figure 2 showed that all four of these proteins were completely coincident with the iTRAQ quantification trend.

### Bioinformatics analysis for the differentially expressed MAL-associated serum glycoproteins

**Figure 3:**
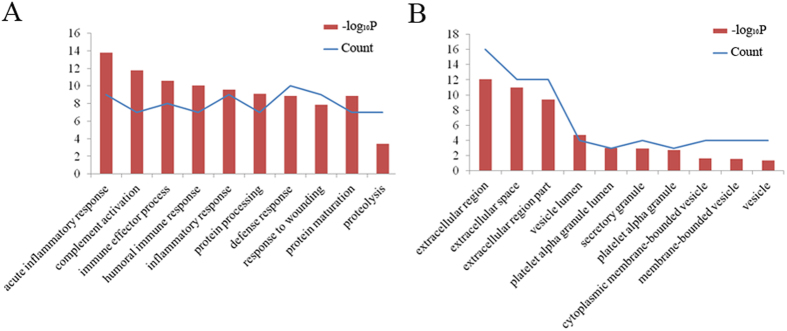
Functional annotation analysis by DAVID. (**A**) The biological processes of the differentially expressed MAL-associated glycoproteins identified by Nano-HPLC-MS/MS analysis. (**B**) The cellular components of the differentially expressed MAL-associated glycoproteins.

To further understand the roles of these differentially expressed MAL-associated serum glycoproteins, DAVID analysis and Ingenuity Pathway Analysis (IPA) analysis were performed. Figure 3A shows that these differential glycoproteins were mainly involved in the acute inflammatory response, complement activation, protein processing, maturation and proteolysis; furthermore, nearly 63% of the differentially expressed MAL-associated serum glycoproteins were involved in the defense response. The cellular component functional annotation of these glycoproteins was mainly enriched in the extracellular region and in the extracellular space (Fig. 3B). In addition, molecular function analysis of the differentially expressed MAL-associated serum glycoproteins indicated that the most common functional annotations of these proteins were inhibitor activity and complement binding (not shown here).

**Figure 4:**
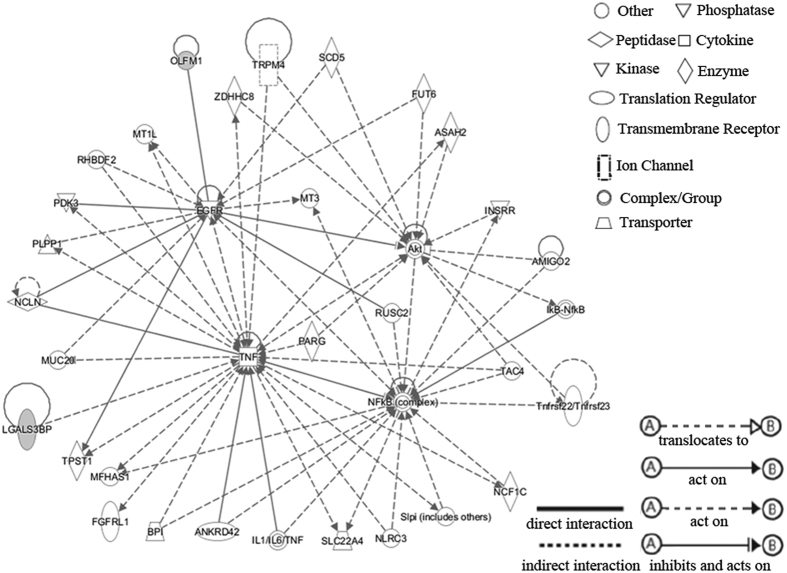
Corresponding network by IPA analysis.

One of the top corresponding IPA networks of the series of 17 differentially expressed MAL-associated serum glycoproteins is shown in Fig. 4. There were 11 network shapes in the network: peptidase, transmembrane receptor, phosphatase, kinase, cytokine, enzyme, complex/group, transporter, ion channel, translation regulator and other. Moreover, four significant nodes, including tumor necrosis factor (TNF), Nuclear factor-kappa B (NFkB), protein kinase B (PKB, also known as Akt) and epidermal growth factor receptor (EGFR), were enriched in the network.

### Analysis of Gal-3BP and MAL-associated Gal-3BP levels in pooled serum samples

**Figure 5:**
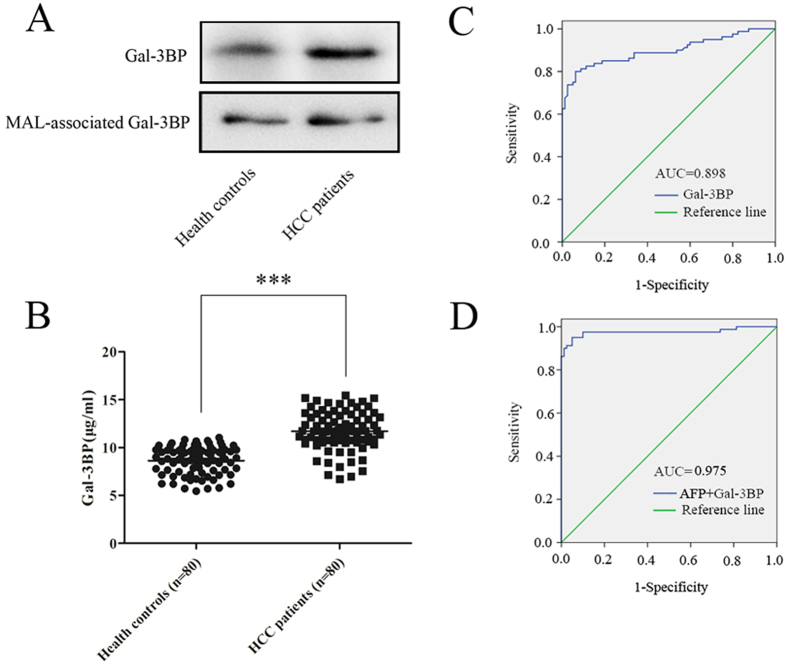
Gal-3BP showed a relative sensitivity in the diagnosis of HCC with chronic HBV infection. (**A**) Gal-3BP was immunoprecipitated in pooled serum samples from 15 early HCC patients with chronic HBV infection and 15 healthy controls, respectively. Lectin blotting detection was performed, and the cropped blots showed that the glycosylation of serum Gal-3BP was highly stable between HCC patients and healthy controls. (**B**) Scatter plot graphs of Gal-3BP intensity by ELISA analysis (serum samples from 80 HCC patients with chronic HBV infection and from 80 healthy controls) were analyzed by Student’s *t*-test, ***p < 0.001. Increased serum Gal-3BP levels in HCC patients with chronic HBV infection were detected. (**C**) ROC curve of serum Gal-3BP level. (**D**) ROC curve of the panel constructed with serum AFP level and serum Gal-3BP level.

Of the three up-regulated MAL-associated proteins, MAL-associated Gal-3BP was selected for further analysis because its expression level was highest in the serum samples. Immunoprecipitation (IP) using Gal-3BP- and MAL-associated Gal-3P-specific antibodies was performed to confirm the expression levels of Gal-3BP and MAL-associated Gal-3BP in pooled serum samples from another group of 15 HBV-related HCC patients and from another group of 15 healthy controls, respectively. Figure 5A showed that more purified Gal-3BP was detected in pooled serum samples from HBV-related HCC patients compared with pooled serum from the healthy controls. Meanwhile, the result from the lectin blotting analysis showed that the proportions of MAL-associated Gal-3BP were similar in equivalent purified serum Gal-3BP samples from both HBV-related HCC patients and healthy controls. In other words, this finding suggests that the up-regulation of MAL-associated Gal-3BP in HBV-related HCC patients compared with the levels of MAL-associated Gal-3BP found in serum from healthy controls was mainly due to the increased expression level of Gal-3BP protein in HBV-related HCC patients but not to significant changes in sialylation in these patients.

### Verification of the clinical diagnostic potential of serum Gal-3BP for HBV-related HCC

The level of Gal-3BP in serum samples from 80 early HCC patients with chronic HBV infection and 80 healthy controls was examined by quantitative ELISA analysis to further explore the clinical diagnostic potential of serum Gal-3BP. Compared with the levels of Gal-3BP in the serum of healthy controls, the levels of Gal-3BP in the serum samples from HBV-related HCC patients were statistically higher (*p* < 0.001, Fig. 5B). The ROC curve was established to evaluate the diagnostic efficacy of the serum Gal-3BP, and the area under the ROC curve (AUC), which can summarize the overall diagnostic accuracy of a potential biomarker, was 0.898 (Fig. 5C). The sensitivity, specificity and accuracy of Gal-3BP to predict HCC were 80.00%, 93.75% and 86.88%, respectively (Table 2).

Considering AFP to be the classical serum biomarker of HCC, the serum level of AFP in these 80 early HCC patients with chronic HBV infection and 80 healthy controls was also examined, and its AUC was 0.901 (Fig. S1); the associated sensitivity, specificity and accuracy of AFP were 87.50%, 93.75% and 90.63%, respectively (Table 2). Then, we constructed a logistic regression model and identified a panel constructed jointly with serum AFP level and serum Gal-3BP level. The predictive model for distinguishing HCC patients from healthy controls was as follow:


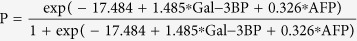


The value 0.3733 was defined as a cut off value (*p* < 0.001); the positive likelihood ratio was 19; and Nagelkerke R was 0.845. Figure 5D shows that the panel provided a higher diagnostic accuracy of HCC, and the AUC was up to 0.975, with a sensitivity of 95.00%, specificity of 95.00% and accuracy of 95.00% (Table 2), which improved 7.5%, 1.25% and 4.37% compared with AFP alone, respectively.

## Discussion

HCC is the second most common malignant cancer in China, and there is a high prevalence of HBV infection among Chinese patients with HCC^18^. Currently, α-1-Fetoprotein (AFP) is mainly used in the clinic for diagnosis of primary HCC. However, because its sensitivity and specificity are not ideal^19^, novel diagnostic biomarkers for the diagnosis of early HBV-related HCC are greatly needed. In recent years, the use of ‘omic’ methods, especially quantitative proteomics analysis, to search for and identify novel HCC biomarkers has attracted extensive attention. For instance, Naboulsi *et al*. demonstrated that versican core protein (VCAN) is a potential biomarker for early HCC diagnosis by label-free discovery analysis: selected (multiple) reaction monitoring (SRM/MRM)^20^. It was also reported that protein S100A9, which was dramatically up-regulated in sera from HCC patients, is a candidate HCC diagnostic biomarker^21^. Lu used the mass spectroscopic approach to characterize the metabolic features of the liver in HCC patients and verified that serum acetylcarnitine was a meaningful biomarker reflecting HCC diagnosis and progression^22^. Our group has focused on potential biomarkers of HCC for several years and reported that heat-shock protein 27 (HSP27) could be a potential biomarker of HCC by 2-DE based on serum proteome analysis^23^. Furthermore, we reported a series of glycoprotein biomarkers of HCC, such as paraoxonase 1 (PON1) and haptoglobin (Hp), through quantitative ELSIA analysis^24^,^25^. In a retrospective study, we performed differential MAL-associated serum glycoproteomics analysis in pooled serum samples from early HCC patients with chronic HBV infection and in pooled samples from healthy controls by MAL affinity chromatography combined with iTRAQ followed by Nano-HPLC-MS/MS analysis. Of these MAL-associated serum glycoproteins, Gal-3BP was further validated as a promising candidate for diagnosing early HCC; this result further confirmed that the combined use of serum Gal-3BP and serum AFP might serve as a potential marker for distinguishing early HCC patients with chronic HBV infection from healthy controls in the Chinese population.

In this study, it was further found that 17 differentially expressed MAL-associated serum glycoproteins were significantly enriched in four significant nodes (TNF, NFkB, Akt and EGFR) of the IPA network, which were all involved in the incidence of HCC. Among them, NFkB can regulate genes for cell adhesion, proliferation, differentiation and apoptosis, and an imbalance in NFkB activity has been associated with the development of HCC^26^. Further, TNF can induce cell death and excessive hepatocyte apoptosis, whereas NFkB can protect hepatocytes against TNF-induced cell death^27^. In addition, diverse lines of evidence have suggested that EGFR can bind transforming growth factor α (TGF-α) and that the activation of the TGF-α/EGFR pathway contributes to HCC formation^28^. It was also proven that blocking the activity of EGFR has an antitumor effect on the development of HCC^29^, whereas the AKT signaling pathway has been firmly established as a major determinant of HCC occurrence and might be the target of specific inhibiting therapies^30^,^31^. Overall, these results suggest that the differentially expressed MAL-associated serum glycoproteins in our study might be involved in the development of HCC by interacting with the significant nodes in the network. However, the direct interactions of these glycoproteins with the significant nodes remain elusive; moreover, the specific mechanisms of how these glycoproteins are involved in HCC development are unknown and will require further research.

Gal-3BP was identified as one of the differentially expressed MAL-associated serum glycoproteins between HBV-related HCC patients and healthy controls in our study; meanwhile its expression quantity was the highest among the three up-regulated MAL-associated proteins. In contrast, although α-2-macroglobulin was more significantly different protein in this study, it had been reported already as a novel cytochemical not serological marker to identify rat hepatocellular preneoplastic and neoplastic lesions^32^,^33^. So we chose MAL-associated Gal-3BP for further analysis on serum samples from HCC patients and healthy controls. Gal-3BP is a secreted protein composed of 585 amino acid residues, and it is widely expressed in many tissues and epithelial cells^34^,^35^. It was previously reported that the serum Gal-3BP levels were elevated in patients with cancer and viral infections, such as hepatitis C virus (HCV)^36^. Difference gel electrophoresis (DIGE) analysis showed a statistically significant increase in the plasma Gal-3BP levels in HCV-infected cirrhotic patients^37^. Other studies demonstrated that there are complex type N-glycans with terminal Siaα2-3 structure on Gal-3BP proteins from breast cancer cells and that as a large hyperglycosylated protein, Gal-3BP could act as a ligand for galectins by glycan-dependent interactions and induce galectin-mediated tumor cell aggregation^38^. Of note, it was found and confirmed that the serum Gal-3BP levels were significantly elevated in HCC patients in comparison to those of healthy individuals^39^, consistent with our report. Furthermore, in our study, the serum Gal-3BP level provided a relatively high diagnostic accuracy for distinguishing HBV-related HCC patients in the Chinese population, and based on the predictive model, the combined use of serum Gal-3BP and serum AFP could improve the sensitivity, specificity and accuracy in the diagnosis of HCC. Furthermore, the serum Gal-3BP level also showed a relative sensitivity in the diagnosis of HCC, even with low AFP levels (not shown here). These results suggested that the combined use of serum Gal-3BP and serum AFP might make up for the low sensitivity of serum AFP alone in the diagnosis of HCC.

However, there are some limitations to this study. Although it was also found that the sialylation of Gal-3BP in serum samples was relatively stable between patients with and without HCC, this study cannot rule out a possible role of the glycosylation of Gal-3BP in regulating the structure and function of the glycoprotein and how it takes part in the development of HCC; the role of glycosylation in the regulation of Gal-3BP in HCC is thus worthy of further investigation. Moreover, further validation in larger samples is needed to confirm that the model constructed by combining serum Gal-3BP with serum AFP could aid in the diagnosis of HCC superior to AFP alone.

In conclusion, we acquired the MAL-associated serum glycoproteomics profile of Chinese HCC patients with chronic HBV infection and healthy controls by iTRAQ combined with Nano-HPLC-MS/MS analysis, and we demonstrated that serum Gal-3BP is a promising diagnostic candidate of HBV-related HCC by quantitative ELISA analysis. Moreover, this is the first report that, based on a predictive model, the combined use of serum Gal-3BP and serum AFP might be a potential marker to identify HBV-related HCC patients.

## Methods

### Clinical specimens

In this study, serum samples from 110 Chinese early HCC patients with chronic HBV infection (The TNM Classification of Malignant Tumours (TNM) was I and II) and from 110 healthy controls were collected at the First Affiliated Hospital of Guangxi Medical University (Guangxi, China) and stored at −80 °C for further analyses. The general information and clinical characteristics of these HCC patients and healthy controls are shown in Table 3. Pooled serum samples from 15 early HCC patients with chronic HBV infection and from 15 healthy controls were used for identifying MAL-associated serum glycoprotein profiles by MS, and other pooled serum samples from 15 HBV-related HCC patients and from 15 healthy controls were used to re-identify the results from MS by western blotting. Additionally, serum samples from 80 HBV-related HCC patients and from 80 healthy controls were used in further quantitative ELISA analyses. Informed consent was obtained from each patient; all of the projects in the study were approved by Ethics Committee of the First Affiliated Hospital of Guangxi Medical University, and all methods were performed in accordance with the human experimentation guideline of the People’s Republic of China.

### Lectin affinity chromatography and iTRAQ labeling

MAL-agarose was washed and supplemented with lectin-binding solution (10 mM Tris-HCl, 0.15 M NaCl, 1 mM CaCl2, 1 mM MgCl2, pH 7.5). The same volume of serum from HBV-related HCC patients and from healthy controls was processed using a ProteoExtract^®^ Albumin/IgG removal kit (Calbiochem, Billerica, MA, USA) to deplete albumin and IgG; the samples were then added into MAL-agarose and incubated at 4 °C overnight. Next, the agarose was washed with lectin-binding solution, and bound fraction was eluted with 20 mM ethylenediamine according to a previously reported protocol^40^. The eluted fractions were labeled with iTRAQ^TM^ Reagent Kit (Applied Biosystems, Waltham, MA, USA): the eluted fractions from serum samples from Chinese HCC patients with chronic HBV infection were labeled with the 119 isobaric tag, and the eluted fractions from serum samples from healthy controls were labeled with the 121 isobaric tag; the flow chart of iTRAQ analysis was summarized in Fig. S2.

### Nano-HPLC-MS/MS analysis

The Nano-HPLC MS/MS analysis was carried out according to previous studies^41^,^42^ by a Nano Aquity UPLC system (Waters Corporation, Milford, MA, USA) coupled with a quadrupole-Orbitrap mass spectrometer (Q-Exactive, Thermo Fisher Scientific, Bremen, Germany) equipped with an online nano-electrospray ion source. The protocol was described as follows: each sample was resuspended in 0.1% formic acid and loaded onto a trap column (Thermo Scientific Acclaim PepMap C18, 100 μm × 2 cm) for 3 min at a flow rate of 10 μl/min. The sample subsequently separated by the analytical column (Acclaim PepMap C18, 75 μm × 25 cm) with a linear gradient, from 5–30% phase B in 95 min (phase A: water with 0.1% formic acid; phase B: ACN with 0.1% formic acid). The flow rate of the analytical column was maintained at 300 nL/min, and the column temperature was maintained at 45 °C. The Q-Exactive mass spectrometer was operated in data-dependent mode with full-scan MS spectra from 350–1600 m/z, resolution at 70 K, followed by fifteen sequential high energy collisional dissociation (HCD) MS/MS scans with a resolution of 17.5 K. In all cases, one microscan was recorded using dynamic exclusion of 30 seconds.

The raw mass spectrometry data files were extracted using the Proteome Discoverer software (Thermo Fisher Scientific, version 1.4.0.288), and the MS/MS results were analyzed using Mascot (Matrix Science, London, UK; version 2.3). The search parameters were as follows: Database was specified in Mascot as Uniprot-SwissProt (Taxonomy: human, 20201 entries), enzyme was specified as trypsin, a fragment ion mass tolerance was specified as 0.050 Da and a parent ion tolerance was specified as 10.0 PPM. Carbamidomethyl of cysteine and iTRAQ 8-plex of lysine and the N-terminus were specified in Mascot as fixed modifications, and Oxidation of methionine and iTRAQ 8-plex of tyrosine were specified as a variable modification. Peptide level false discovery rates (FDR) were controlled lower than 1% by the percolator algorithm. Protein quantifications were performed by unique peptides, and experimental bias was corrected by the method of normalization on the protein median; the minimum number of proteins that must be observed to allow normalization was set to 100.

### Western blotting

Equivalent volumes of eluted MAL-associated fraction in pooled albumin- and IgG-depleted serum samples from Chinese HBV-related HCC patients and from healthy controls were separated by SDS-PAGE and analyzed by western blotting. In brief, proteins in the gels were transferred onto PVDF membranes (Millipore, Billerica, MA, USA). After blocking for nonspecific binding, the membranes were incubated with antibodies against α-2-macroglobulin, α-1-antitrypsin, Gal-3BP and vitamin D-binding protein (R&D, Minneapolis, MN, USA) at 4 °C overnight, respectively. Then, the membranes were washed with 0.1% TBS-Tween20 (TBST, 50 mM Tris, 150 mM NaCl, 0.1% Tween 20, pH 7.6), and incubated with HRP-conjugated secondary antibodies for 1 h at room temperature (Kangcheng, Shanghai, China). After the membranes were washed three times with TBST, Amersham^TM^ ECL^TM^ prime western blotting detection reagents (GE Healthcare, Piscataway, NJ, USA) were used to detect the bands on the membranes.

### Bioinformatics analysis

The identified differentially expressed MAL-associated proteins were evaluated using the Uniprot database (http://www.uniport.org). Further functional categories analysis was performed by the Database for Annotation, Visualization and Integrated Discovery (DAVID, https://david.ncifcrf.gov/), and Ingenuity Pathway Analysis (IPA, QIAGEN, Redwood City, CA, USA) was used to investigate the biological interactions of them.

### Immunoprecipitation and lectin blotting

Albumin- and IgG-depleted serum sample proteins were immunoprecipitated with Gal-3BP antibody using Pierce^®^ Co-Immunoprecipitation Kit according to the recommendation of the manufacturer. Equivalent volumes of eluted immune-complexes were run on 10% SDS-PAGE gels and incubated with Gal-3BP antibody to perform the western blotting detection as described above. Subsequently, the sample amounts were adjusted according to the quantitative analysis of immunoblotted bands, and the PVDF membranes were incubated with biotinylated MAL (Vector Laboratories, Burlingame, CA, USA) and Streptavidin Horseradish Peroxidase (HRP) Conjugate (Invitrogen, Waltham, MA, USA) in turn to perform lectin blotting detection.

### ELISA analysis

Levels of Gal-3BP protein in serum samples from HBV-related HCC patients and healthy controls were assayed by Human Gal-3BP Quantikine^®^ ELISA kits (R&D, Minneapolis, MN, USA) according to the recommendation of the manufacturer. Serum samples were diluted (1:500) in sample diluents, respectively before the assay.

### Statistical analysis

Statistical analysis was performed by SPSS 16.0 statistical packages (SPSS Inc., Chicago, IL, USA). Data were presented as the mean ± SD, unless otherwise indicated. Quantitative variables were evaluated using Student’s *t*-test (two tailed) to compare two groups of parametric variants; *p* < 0.05 was considered statistically significant. Receiver operating characteristic (ROC) curves generated by sensitivity and 1-specificity were used to determine the diagnostic value of biomarkers; the cutoff was defined as the point in the ROC curve that maximizes the value of sensitivity plus specificity.

## Additional Information

**How to cite this article**: Liu, T. *et al*. Discovering potential serological biomarker for chronic Hepatitis B Virus-related hepatocellular carcinoma in Chinese population by MAL-associated serum glycoproteomics analysis. *Sci. Rep.* **7**, 38918; doi: 10.1038/srep38918 (2017).

**Publisher's note:** Springer Nature remains neutral with regard to jurisdictional claims in published maps and institutional affiliations.

## Supplementary Material

Supplementary Information

## Figures and Tables

**Table 1:** Differentially expressed MAL-associated serum glycoproteins between Chinese HCC patients with chronic HBV infection and healthy controls.

Accession	Gene Symbol	Name	Peptides	121/119
P01023	A2MG	Alpha-2-macroglobulin	2	3.576
P01024	CO3	Complement C3	1	1.428
P01009	A1AT	Alpha-1-antitrypsin	10	1.409
P01871	IGHM	Ig mu chain C region	9	1.358
P05155	IC1	Plasma protease C1 inhibitor	43	1.270
Q08380	LG3BP	Galectin-3-binding protein	23	1.253
P0C0L5	CO4B	Complement C4-B	7	1.250
P0C0L4	CO4A	Complement C4-A	1	1.230
P05090	APOD	Apolipoprotein D	1	0.794
Q99784	NOE1	Noelin	1	0.790
P06888	LV109	Ig lambda chain V-I region EPS	1	0.788
P02760	AMBP	Protein AMBP	12	0.782
P09172	DOPO	Dopamine beta-hydroxylase	22	0.774
P00751	CFAB	Complement factor B	8	0.761
P02774	VTDB	Vitamin D-binding protein	1	0.750
P02745	C1QA	Complement C1q subcomponent subunit A	4	0.723
P06681	CO2	Complement C2	15	0.700

**Table 2:** Diagnosis values of serum AFP level and serum Gal-3BP level in differentiating HCC patients with chronic HBV infection from healthy controls.

Biomarker	AUC	Sensitivity	Specificity	Accuracy
AFP	0.901	87.50%	93.75%	90.63%
Gal-3BP	0.898	80.00%	93.75%	86.88%
AFP + Gal-3BP	0.975	95.00%	95.00%	95.00%

**Table 3:** General information and clinical characteristics of Chinese HCC patients with chronic HBV infection and healthy controls^a^.

Group^b^	Healthy controls	HCC patients
Sample size	110	110
Age (year)	49 ± 11	51 ± 13
Gender (male/female)	85 (77.27%)/25 (22.73%)	87 (79.09%)/23 (20.91%)
AFP (μg/L)	3.72 (1.37–9.62)	7836.37 (1.74–60500.00)
ALT (U/L)	21.32 (12–49)	53.81 (24–481)
AST (U/L)	22.73 (15–40)	63.98 (18–300)
HbsAg (yes/no)	0 (0.00%)/110 (100.00%)	110 (100%)/0 (0%)
TNM	/	I (n = 57), II (n = 53)

^a^The values supplied in Table 3 were means with SD or range.

^b^Abbreviations: AFP, alpha fetoprotein; ALT, alanine aminotransferase; AST, aspartate transaminase; HbsAg, hepatitis B surface antigen.
