# Supplementary material for: Discovering potential serological biomarker for chronic Hepatitis B Virus-related hepatocellular carcinoma in Chinese population by MAL-associated serum glycoproteomics analysis
Source: Sci Rep. 2017 Jan 12;7:38918. doi: 10.1038/srep38918 (PMC5228127; doi:10.1038/srep38918)

Discovering potential serological biomarker for chronic Hepatitis B Virus-related hepatocellular carcinoma in Chinese population by MAL-associated serum glycoproteomics analysis

Tianhua Liu [1], Denghe Liu [2], Riqiang Liu [3], Hucong Jiang[1], Guoquan Yan[1], Wei Li [1], Lu Sun [1], Shu Zhang [1], Yinkun Liu [1], Kun Guo [1]*

1 Liver Cancer Institute, Zhongshan Hospital, Fudan University, Key Laboratory of Carcinogenesis and Cancer Invasion, Ministry of Education, Cancer Research Center, Institutes of Biomedical Sciences, Fudan University, Shanghai, China.

2 Department of Clinical Laboratory, First Affiliated Hospital of Guangxi Medical University, Nanning, Guangxi, China.

3 People's Hospital of Gangxi Zhuang Autonomous Region, Nanning, Guangxi, China.

Correspondence to: Guo Kun, guo.kun@zs-hospital.sh.cn

Tel: +86- 21-54237963;

Fax: +86-21-54237959;

Additional file

Additional file 1: Figure S1. The flow chart of iTRAQ labeling.

Additional file 2: Figure S2. ROC curve of serum AFP level.

Figure S1

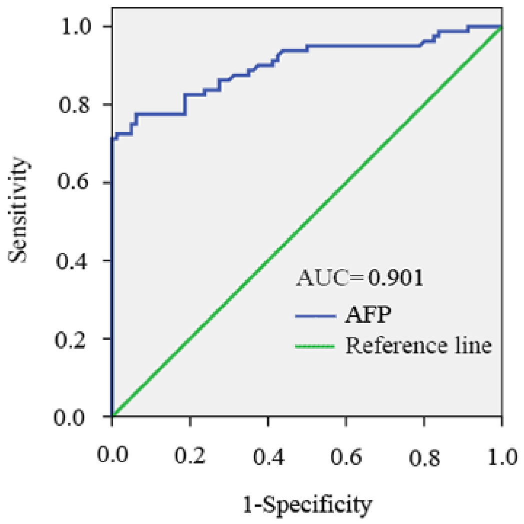

Figure S2

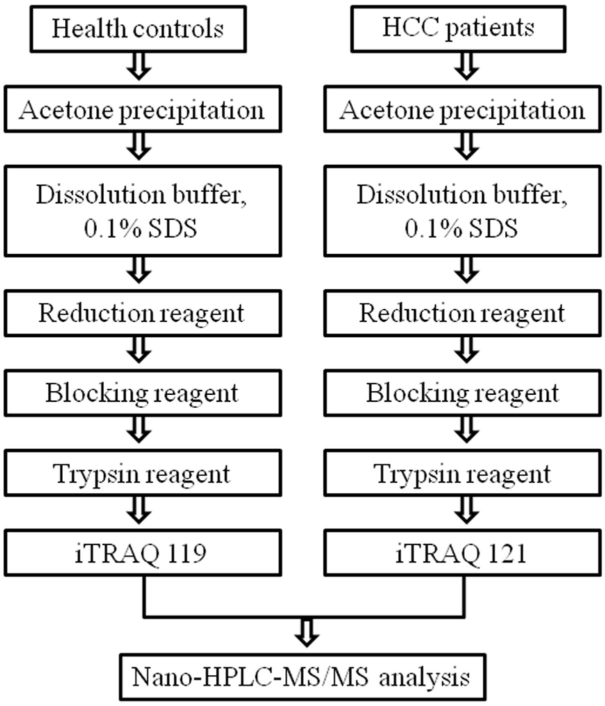

Supplementary information file of full-length blots

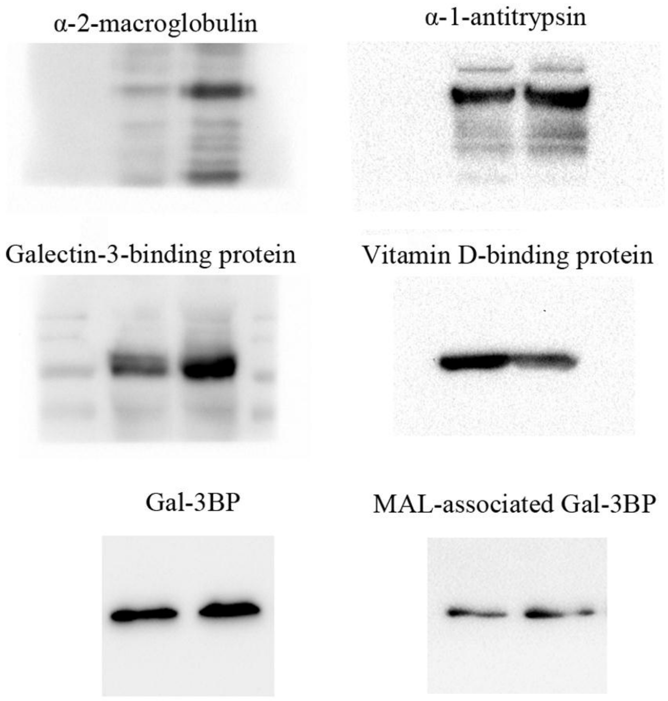

Supplement: Supplementary Information [file srep38918-s1.pdf]
